# Comparative efficacy of moxibustion as an add-on treatment with different durations for diabetic peripheral neuropathy: study protocol for a randomized controlled trial

**DOI:** 10.3389/fneur.2025.1609674

**Published:** 2025-08-29

**Authors:** Yurong Kang, Hantong Hu, Can Lin, Yu Zheng, Minjian Jiang, Zhouyuan Wei, Xiaofen He, Jianqiao Fang, Yongliang Jiang

**Affiliations:** ^1^Zhejiang Chinese Medical University, Hangzhou, China; ^2^Key Laboratory of Acupuncture and Neurology of Zhejiang Province, Department of Neurobiology and Acupuncture Research, The Third Clinical Medical College, Zhejiang Chinese Medical University, Hangzhou, China; ^3^The Third Affiliated Hospital of Zhejiang Chinese Medical University, Hangzhou, Zhejiang, China

**Keywords:** diabetic peripheral neuropathy, pain, moxibustion, randomized controlled trial, protocol

## Abstract

**Background:**

Diabetic Peripheral Neuropathy (DPN) can markedly diminish patients’ quality of life. Current treatments provide limited relief, driving interest in non-invasive options such as moxibustion. Moxibustion, a technique rooted in acupuncture, shows promise for managing pain. However, it lacks standardized protocols for treating DPN, especially concerning moxibustion duration, and its effectiveness for DPN is not well-supported by evidence. Thus, this study seeks to identify the optimal moxibustion duration to relieve DPN symptoms and enhance nerve function, filling an important gap in clinical practice.

**Methods:**

Participants will be randomly allocated to three clinical centers, with 30 individuals at each center, and evenly divided among the conventional treatment group, the 15-min moxibustion group, and the 30-min moxibustion group. The conventional treatment group will be administered mecobalamin and epalrestat for a duration of 4 weeks, while the moxibustion groups will receive moxubustion as an add-on therapy treatment twice a week over the same period. The duration of moxibustion differs from the 15-min group, while the procedure remains consistent across the moxibustion groups. The primary outcome is total clinical effectiveness. The second outcomes include electrophysiological examination, the Toronto Clinical Scoring System (TCSS), the Visual Analogue Scale (VAS), the Traditional Chinese Medicine Syndrome Score Scale (TCMS), and infrared thermography testing. The outcomes will be assessed during the baseline period, after the 8th treatment, and at the one-month follow-up.

**Conclusion:**

This trial aims to identify the optimal moxibustion duration for DPN symptom relief and nerve function improvement, offering evidence for standardized clinical protocols. The findings could enhance treatment efficacy, reduce adverse effects, and alleviate DPN’s socio-economic burden.

**Clinical trial registration:**

https://clinicaltrials.gov/, NCT06330233.

## 1 Introduction

Diabetic peripheral neuropathy (DPN) is one of the most common chronic complications of diabetes. Epidemiological survey results suggest that diabetes ranks as the third chronic non-communicable disease posing a significant threat to human health, following tumors and cardiovascular and cerebrovascular diseases (1, 2). By 2030, it is projected that the global population of people with diabetes will reach 643 million, with China currently having 114 million adult diabetic patients or 24% of the worldwide diabetic population (1). The primary symptoms of DPN are numbness, paresthesia, freezing and burning sensations, pain, and other reactions in the distal feet and toes, resulting in significant physical and emotional distress for patients (3, 4).

Currently, western treatment for DPN is limited in its ability to alleviate clinical symptoms (5–7) and frequent adverse effects., hence elevating the risk of impairment and significantly impacting patients’ quality of life (8). Moreover, these pharmacological interventions often necessitate prolonged use, increasing treatment burden and posing challenges to patient adherence. Consequently, these shortcomings underscore an urgent need for optional, safe, and effective therapeutic strategies to enhance clinical outcomes in DPN.

Acupuncture therapy is a traditional Chinese medical practice that entails the insertion of needles into certain points on the body to stimulate and treat various diseases (9–11). From an evidence-based perspective, acupuncture can modulate the nervous system and other physiological processes to produce theraputic effects like pain relief (12–15). A meta-analysis reveals that acupuncture, when combined with conventional treatment, successfully alleviates discomfort associated with DPN (16). Our early research concentrated on the fundamentals of acupuncture analgesia (17, 18), as well as a clinical study on the difference in moxibustion-induced microcirculatory responses in different meridians (19). In terms of clinical practice, moxibustion is similar to acupuncture, can treat DPN, considerably improve patients’ symptoms, and has few side effects (16, 20). Moxibustion is a supplementary and alternative therapy in which mugwort is burned at certain acupoints to offer warming stimulation for treatment and recovery (21, 22). From an evidence-based viewpoint, moxibustion potentially exerts effects through thermal stimulation that may influence local circulation and neural pathways (23–25). Moxibustion on appropriate acupoints in DPN patients can successfully improve local neurotrophic symptoms, improve nerve cell function, boost sensitivity and nerve conduction velocity, and have significant therapeutic effects (19, 26).

Emerging clinical evidence suggests that moxibustion can alleviate DPN-associated pain and numbness (20). However, its therapeutic protocols remain unstandardized, particularly with respect to treatment duration. Existing studies report a wide range of moxibustion durations—from a few minutes to as long as 30 min—with little consensus on the optimal timeframe or its impact on efficacy. This gap in knowledge hinders the broader adoption and optimization of moxibustion as a viable treatment for DPN.

Therefore, we hypothesize that the combination of moxibustion and conventional treatment for diabetic peripheral neuropathy is more efficacious than conventional treatment alone. Nonetheless, the distinction in efficacy between the 15-min moxibustion group and the 30-min moxibustion group necessitates further investigation.

To address this critical research gap, this randomized controlled trial aims to evaluate the effects of varying moxibustion durations (15 min vs. 30 min) on symptom relief and nerve function improvement in patients with DPN. By identifying the optimal duration of moxibustion, this research seeks to provide evidence-based guidance for the clinical management of DPN and advance the standardization of moxibustion therapy.

## 2 Methods and analysis

### 2.1 Study design

**Figure 1:**
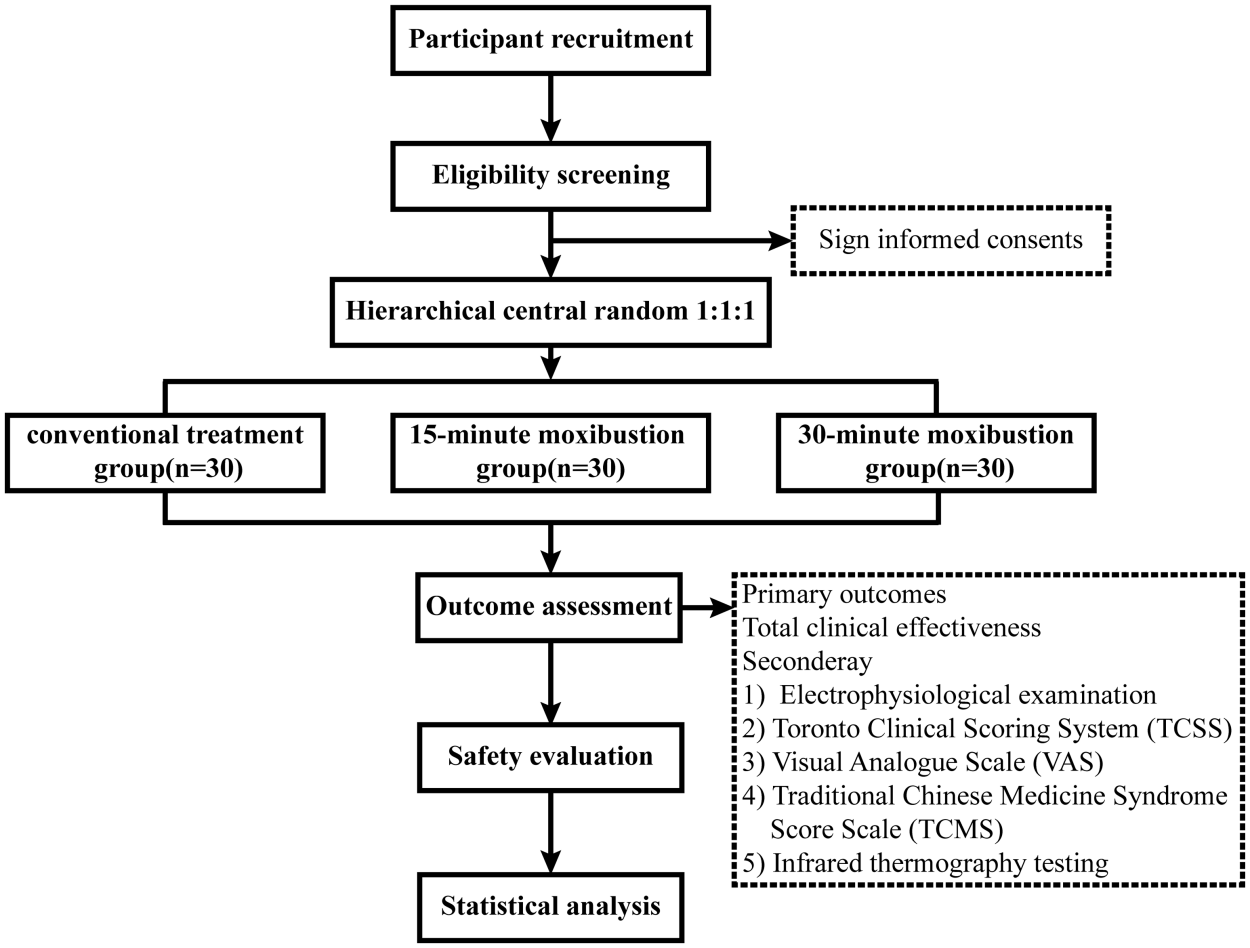
Flow chart of the study process.

This trial is a multi-clinical center, open method, randomized controlled trial (RCT), three-arm, parallel-group design. The study is designed to assess moxibustion as an add-on therapy to conventional treatment. This study protocol obtained approval from the Ethics Committee of the Third Hospital Affiliated with Zhejiang University of Traditional Chinese Medicine and has been registered with the Clinicaltrials registry (identification code: NCT06330233). This trial protocol is developed in accordance with the Standard Protocol Items: Recommendations for Interventional Trials (SPIRIT) guideline. The schedule of this trial is presented in Table 1, and the flow diagram of the study is shown in Figure 1.

**Table 1:** Schedule of enrolment, interventions and outcome assessments.

Study period	Baseline	Treatment phase (4 weeks)		Follow-up phase (1 month)
The time point	Week 0	Week 2	Week 4	month 1
Eligibility screening	√			
Demographic information	√			
Case data	√			
Inclusion criteria	√			
Exclusion criteria	√			
Moxibustion treatment		√	√	
Outcomes assessment				
(1) Total clinical effectiveness			√	√
(2) Electrophysiological examination	√		√	
(3) TCSS	√		√	√
(4) VAS	√		√	√
(5) TCMS	√		√	√
(6) Infrared thermography testing	√		√	
Safety evaluation		√	√	

√, required; TCSS, Toronto Clinical Scoring System; VAS, visual analog scale; TCMS, traditional Chinese medicine syndrome score scale.

### 2.2 Participant recruitment

Eligible DPN patients will be recruited from the Third Affiliated Hospital of Zhejiang Chinese Medical University, the First Affiliated Hospital of Zhejiang Chinese Medical University, and the Zhejiang Greentown Cardiovascular Hospital. Each participant will be provided with a detailed procedure description before signing the informed consent form.

### 2.3 Diagnosis criteria

The diagnostic criteria for DPN, established in accordance with Chinese recommendations (27), are delineated as follows.

- A clear history of Type 2 diabetes mellitus.
- Peripheral neuropathy that appeared at the time of or after the diagnosis of diabetes mellitus.
- Clinical symptoms (pain, numbness, sensory abnormalities, etc.) and signs are consistent with the presentation of DPN.
- Abnormalities in any of the 5 tests (ankle reflex, pinprick nociception, vibration sensation, pressure sensation, temperature sensation).
- Or in the absence of clinical symptoms, any two of the 5 tests are abnormal.
- Rule out other diseases that cause peripheral neuropathy. Identify neurotoxic effects caused by drugs, especially chemotherapeutic drugs, and neurological damage caused by metabolic poisons due to renal insufficiency.

### 2.4 Inclusion criteria

- History of diabetes mellitus.
- Persistent pain and/or sensory abnormalities in the extremities (at least in both lower limbs), decreased bilateral or unilateral ankle reflex, decreased vibration sensation (medial ankle is weaker than medial tibial condyle), decreased nerve conduction velocity on the affected side, TCSS score > 5.
- 18 years ≤ age ≤ 80 years.
- Gender is not limited.
- Those who have the ability of independent in daily life and can cooperate to complete all the examinations.
- Those who are conscious without serious mental diseases and cognitive disorders, and those who do not have serious cardiac, cerebral, hepatic, renal, and other internal diseases.
- Voluntary participation and signing of informed consent.

### 2.5 Exclusion criteria

- Those with peripheral neuropathy, ulcers, and gangrene of the limbs caused by various other reasons (e.g., hypothyroidism, alcohol, drugs, heredity, etc.), or those with a history of skin ulcers or lesions that do not heal easily.
- Women who are in preparation for pregnancy, during pregnancy, or breastfeeding.
- Those with acute complications such as combined diabetic ketoacidosis, lactic acidosis, severe infections, etc.
- Those who suffer from serious liver or kidney damage or serious cardiovascular and cerebrovascular diseases (angina pectoris, myocardial infarction, multiple cerebral infarctions, cerebral hemorrhage, etc.).
- Those who have scars or pigmentation on the skin of the testing site, will affect the accuracy of the test.
- Those who are participating in other acupuncture or drug clinical trials.

### 2.6 Withdrawal criteria

- If adverse drug reactions, acupuncture-related adverse events, significant physiological changes, or severe complications or deterioration of the condition arise during the research process, and a professional physician evaluates that continued participation is inadvisable, the researcher must terminate the study.
- The patient is noncompliant and refuses to adhere to the therapy regimen.
- Participant voluntarily requests to withdraw.

Researchers must diligently record the rationale and timing of individuals’ removal from the trial. Individuals who have undergone more than half of the treatment regimen (≥4 sessions) should be incorporated into the efficacy analysis.

### 2.7 Randomization and allocation concealment

Participants eligible for inclusion will be randomly assigned to three groups: the conventional treatment group, the moxibustion 15-min group, and the moxibustion 30-min group. An impartial administrator, not engaged in other study operations, will carry out this process, and the details of the random allocation list will remain absolutely confidential. The random allocation sequence will be computer-generated with a 1:1:1 ratio using the Electronic Data Capture System of Zhejiang Chinese Medical University.

### 2.8 Blinding

This study will not employ blinding for administrators and patients across different moxibustion groups due to the particular characteristics of moxibustion manipulation. However, this study will be blind to the group assignment of outcome assessors made by a specifically designated person.

### 2.9 Intervention procedures

All recruited patients will receive diabetes mellitus education tailored to their conditions and will manage their blood glucose levels through diet, exercise, oral hypoglycemic agents, and insulin administration. Patients with combined hypertension and hyperlipidemia will receive daily treatments aimed at lowering blood pressure and blood lipids, tailored to their specific conditions, to maintain normal control. The choice of medication will be based on the current medications the patients are independently using. The selection of medication must be determined by the patient’s existing self-medication practices.

#### 2.9.1 Conventional treatment group

Mecobalamin tablets (0.5 mg/dose, 3 times/day) and epalrestat (0.5 g/dose, 3 times/day) will be administered orally for 4 weeks in conjunction with the patient’s daily treatment (basal medication treatment for patients with combined hypertension and hyperlipidemia).

#### 2.9.2 Moxibustion groups

Both moxibustion groups will receive the same conventional treatment (mecobalamin and epalrestat) as the control group, with moxibustion as an add-on therapy. In the 15-min moxibustion group, the selection of acupoints is based on traditional Chinese medicine literature (28), moxa cones will be pasted on the acupoints, and moxibustion will be applied by ignition, each time for 15 min at each acupoint, once every three days, twice a week, four consecutive weeks of treatment. To ensure standardization, the moxa cone will be kept at a distance from the skin that elicits a warm sensation without causing pain or burning, and this distance will be consistently maintained by the practitioner.

The 30-min moxibustion group will follow the same operation as the 15-min moxibustion group apart from the intervention duration.

#### 2.9.3 Acupoint selection

Based on traditional Chinese medicine literature (28) and clinical experience, a prescription was developed for the following acupoints, adhering to the concepts of local and distal point selection: Zusanli (ST36), Sanyinjiao (SP06), Hegu (LI4), Quchi (LI11). The above acupoints will be on the affected side. The locations of the above acupoints are summarized in Table 2.

**Table 2:** Locations of the selected acupoints for diabetic peripheral neuropathy treatment.

Acupoint	Location
Zusanli (ST36)	3 cun below the patella, one fingerbreadth lateral to the anterior border of the tibia
Sanyinjiao (SP6)	3 cun above the medial malleolus, on the posterior border of the medial aspect of tibia
Hegu (LI4)	On the dorsum of the hand, between 1st and 2nd metacarpal bones, in the middle of the 2nd metacarpal bone on the radial
Quchi (LI11)	When elbow is flexed with the angle of 90°, the point is at the midpoint of the lateral end of the transverse cubital crease and the lateral epicondyle of the humerus

#### 2.9.4 Concomitant care and intervention

During the study period, participants must avoid other DPN interventions, including behavioral therapies and medications, that could influence the study’s outcomes. Participants will be excluded if they receive additional combined interventions for the treatment of DPN. Participants with comorbid conditions, including hypertension, diabetes, and other chronic diseases, may maintain their standard medications and therapies. Research personnel will document the names of comorbid conditions, medications, and therapies in the case report form.

### 2.10 Outcome measures

#### 2.10.1 Primary outcome

- Total clinical effective rate

Overall clinical effectiveness will be assessed at 2 time points: at week 4 and during the follow-up period (month 1). These specific time points are chosen to evaluate the immediate therapeutic effects following the completion of the 4-week treatment course and to determine the short-term sustainability of these effects 1 month after the intervention has ceased. It will be classified into the following 3 grades according to the Guidelines for Clinical Study of New Chinese Medicines:

1. Cure: clinical symptoms such as limb numbness, coldness and chilling pain, sensory abnormalities, muscle weakness, and muscle atrophy disappear, tendon reflexes return to normal, and the total score of TCSS is ≤ 5 points;
2. Effective: numbness of the limbs, cold and chilling pain, sensory abnormalities, muscle weakness myasthenia, and other clinical symptoms significantly improved, tendon reflexes returned to normal, TCSS score level 2 or 1 level lower;
3. Ineffective: clinical symptoms did not improve or worsen, tendon reflexes were not elicited, and TCSS score grade did not decrease or increase.

Total effective rate = [(number of cured cases + number of effective cases)/total number of cases] × 100%.

#### 2.10.2 Secondary outcomes

- Electrophysiological examination

The electrophysiological examination will be detected at week 0 and after 4 weeks of treatment (week 4). The latency, amplitude, motor nerve conduction velocity (MNCV), and sensory nerve conduction velocity (SNCV) of the tibial nerve and peroneal nerve of the lower extremities will be measured before and after treatment.

- Toronto Clinical Scoring System (TCSS)

The TCSS is a valid instrument to reflect the presence and severity of diabetic peripheral sensorimotor polyneuropathy as measured by sural nerve morphology and electrophysiology (29). TCSS will be estimated at week 0, week 4, and month 1.

- Visual Analogue Scale (VAS)

The level of pain at the subject’s lesion site will be determined using the VAS scale, which classifies pain on a scale of 0–10, with the greater the number the more pronounced the pain, i.e., a score of 0 indicates no pain, 1–3 represents mild pain, 4–6 represents moderate pain, 7–9 represents severe pain, and 10 represents severe pain. Participants will be allowed to indicate the level of pain by crossing out specific numbers on the scale according to their actual situation. The VAS scale will be administered at week 0, week 4, and month 1.

- Traditional Chinese Medicine Syndrome Score Scale (TCMS)

The evaluation of the curative effect of TCMS should refer to the guiding principles of clinical research of new drugs of traditional Chinese medicine and the clinical evidence-based practice guide of traditional Chinese medicine for diabetes. TCMS will be estimated at week 0, week 4, and month 1.

The specific records are as follows:

1. The VAS score of limb pain was consistent according to the severity.
2. The three main traditional Chinese medicine syndromes of limb numbness, abnormal chill (or fever), and ant feeling were scored as 0, 1, 2, and 3 points, respectively, according to their severity.

- Infrared thermography

The test sites will be bilateral plantar, dorsal, palmar, and dorsal hands. The instrument used is the NECR450 infrared thermal imager produced by NECAVIO Company in Japan. Infrared thermography testing will be performed once at week 0 and once at week 4.

#### 2.10.3 Statistical analysis

Statistical analysis of the data will be performed by a third-party statistician who will not involved in the pre-test, using the statistical software IBM SPSS Statistics version number (SPSS 26.0). Measurement information to meet the normal distribution of the use of the mean ± standard deviation table, counting information is expressed in the number of cases (%). The non-parametric rank sum test was employed for group comparisons, while chi-square analysis will facilitate additional group comparisons. Furthermore, a mixed linear model, COX regression analysis, and logistic regression analysis will be utilized for the exploratory examination of factors influencing the primary outcome indices. For missing data handling, multiple imputation will be employed for primary outcome analyses. A *p*-value of less than 0.05 indicates that the differences are statistically significant. Statistical analyses will be conducted by qualified statisticians. Upon completion of data entry and review, statisticians must finalize the statistical analysis promptly and produce a written report detailing the findings.

#### 2.10.4 Sample size estimation

The sample size was calculated based on the primary outcome, defined as the clinical effectiveness rate. Drawing from prior studies and pilot data, the effectiveness rate for the conventional treatment group was estimated at 60%, while the moxibustion groups were hypothesized to achieve an effectiveness rate of 90%. A two-sided test was adopted with a significance level (*α*) of 0.05 and a statistical power (1-*β*) of 90%, ensuring adequate sensitivity to detect the specified effect size, the calculation yielded a minimum of approximately 24 participants per group. To accommodate an anticipated dropout rate of 20%, the sample size was adjusted upward to 30 participants per group. With three study groups, this results in a total sample size of 90 participants.

#### 2.10.5 Safety assessment

Detailed records of any adverse events will be maintained, including the adverse event’s onset, symptoms, duration, severity, treatment, and disappearance. The incidence rate of adverse events will be calculated by recording the number of patients with adverse reactions and dividing it by the total number of patients. In the event of adverse reactions, clinicians must assess the necessity of trial discontinuation based on patient conditions. Serious cases are to be reported to the project management office of the supporting unit and the ethics committee within 24 h. The safety of moxibustion will be assessed at each treatment session.

#### 2.10.6 Study monitoring and quality control

The research group will establish a standardized operating procedure (SOP) for the assay. One month prior to the official launch of the clinical trial, the research group will conduct a specialized training session to ensure standardized training for all participating researchers. The training will primarily concentrate on the implementation plan and associated SOPs, enabling each clinical researcher to understand the research process and specific implementation details necessary for ensuring the reliability of clinical study conclusions. All observations in the clinical study must be verified and confirmed multiple times to ensure the reliability and authenticity of the data, thereby guaranteeing that all results and conclusions in the clinical research are based on the original data. Specialized personnel will be employed to gather and quantify trial data to mitigate trial bias. A specialized data management firm will be engaged for the management of clinical data. Monthly inspections will be conducted to ensure the quality of clinical studies.

## 3 Discussion

As a consequence of the high prevalence of diabetes, DPN is a condition that not only places a load on individuals in terms of their mental burden and financial burden, but it also places weight on society. Most studies focus on acupuncture and electroacupuncture for the treatment of diabetic peripheral neuralgia in both clinical and basic research (16, 30–34), and there is a paucity of research on moxibustion. However, the use of moxibustion in the treatment of diabetic peripheral vascular disease is generally acknowledged in China, and impressive results have been achieved (35). The mechanism of moxibustion in treating DPN may involve the inhibition of NF-kappa B protein and mRNA expression, alongside the induction of Nrf2 protein and mRNA expression in the sciatic nerve, thereby restoring the balance between NF-kappa B and Nrf2 in rats with DPN (36). Moxibustion, although there is a scarcity of clinical studies owing to its operational instability, shows considerable effectiveness in the treatment of diabetic peripheral neuropathy. Therefore, offering a protocol for moxibustion treatment is significant.

Moxibustion, a highly regarded intervention for diabetic complications in China, enjoys popularity, particularly in the fields of rehabilitation and physiotherapy. As of now, the duration of moxibustion for addressing diabetic neuropathic pain remains uncertain and lacks a suitable and standardized protocol. Due to the operation of moxibustion’s lack of standardization, adverse events come and will bring varying degrees of impact on the health of patients such as necrotizing fasciitis (37). The phenomenon described, which pertains to accidents occurring during the moxibustion process, is relatively common. Conditions such as empyrosis, infection, and allergy may arise during surgery due to the prolonged hypoesthesia experienced by diabetic patients, particularly regarding a reduced sense of temperature (38, 39). In this case, it is essential to establish a clinical standard for moxibustion.

The acupoints for acupuncture and moxibustion are generally quite similar (40). A meta-analysis indicates that acupuncture, when combined with conventional treatment, is effective for alleviating pain in diabetic peripheral neuropathy, especially when acupoints are situated in the limbs (16). Studies indicate that acupuncture, utilized as a complementary therapy for DPN, employs often targeted acupoints like Zusanli (ST36), Sanyinjiao (SP6), Hegu (LI4), Quchi (LI11), and Yanglingquan (GB34), among others (41, 42). ST36, SP6, LI4, and LI11 acupoints will be included in this research plan due to the evidence that diabetic peripheral neuropathy frequently impacts the small blood vessels at the extremities of the limbs and the extant clinical studies (42, 43). From the perspective of traditional Chinese medicine, ST36 and SP6 are associated with the meridians of the spleen and stomach in traditional Chinese medicine. The theory of the spleen and stomach can be used to treat patients with diabetes who have been afflicted with the disease for an extended period and have a deficiency of qi in the spleen and stomach. The hand yangming large intestine meridian is the location of 4LI4 and LI11. The yangming meridian is a meridian that is profuse in blood and qi. It has the potential to enhance the circulation of blood and qi in the meridians. The aforementioned are the proposed acupoint selections for this research strategy.

Our work demonstrates both innovation and strengths in clinical protocol. First, the acupoints were chosen based on previous research on the frequency of acupoint selection in DPN treatment (44), allowing us to make an objective choice. Second, in accordance with the traditional Chinese medical understanding of the pathogenesis of DPN, the treatment principle of warming yang and promoting blood circulation was proposed, leading to a multicenter randomized controlled trial examining the efficacy of moxibustion in treating DPN. While a systematic review by Zhou et al. (16) confirmed the benefits of acupuncture for DPN, our study adds to this by focusing specifically on moxibustion, a distinct thermal therapy whose optimal parameters are not yet established. A comparable protocol conducted to assess the efficacy and safety of moxibustion therapy for diabetic peripheral neuropathy does not address the varying durations of moxibustion treatment (21). Our protocol will examine the effectiveness and differences of moxibustion with varying durations in treating DPN and identify the optimal duration for this treatment.

Some limitations remain associated with this investigation. Firstly, unlike other studies, we cannot employ blinding due to the inherent characteristics of the moxibustion procedure. Secondly, this study uses the Visual Analogue Scale (VAS) to assess pain intensity. While widely validated for general pain, it does not specifically capture the distinct qualities of neuropathic pain. The inclusion of a dedicated neuropathic pain scale, such as the Leeds Assessment of Neuropathic Symptoms and Signs Scale (LANSS), would have provided a more nuanced assessment, and this should be considered in future research. Thirdly, the study will be conducted exclusively in Chinese populations, which may limit the generalizability of findings to other ethnic groups. Additionally, the relatively short follow-up period (1 month) may not capture long-term treatment effects or potential delayed responses to moxibustion therapy.

## 4 Conclusion

This study protocol aims to determine the optimal duration of moxibustion treatment for diabetic peripheral neuropathy through a rigorous multicenter randomized controlled trial design. By comparing 15-min and 30-min moxibustion durations as add-on therapies to conventional treatment, this research will provide evidence-based guidance for clinicians and contribute to the standardization of moxibustion protocols for DPN management. The expected outcomes include improved clinical effectiveness rates, enhanced nerve function parameters, and reduced neuropathic pain, which collectively may lead to better quality of life for DPN patients and reduced healthcare burden.

## References

[1] Ho LJ Sheu WH Lo SH Yeh YP Hwu CM Huang CN . Unhealthy lifestyle associated with increased risk of macro- and micro-vascular comorbidities in patients with long-duration type 2 diabetes: results from the Taiwan diabetes registry. Diabetol Metab Syndr. (2023) 15:38. doi: 10.1186/s13098-023-01018-9, PMID: 36890551 PMC9996995

[2] Charnogursky G Lee H Lopez N. Diabetic neuropathy. Handb Clin Neurol. (2014) 120:773–85. doi: 10.1016/B978-0-7020-4087-0.00051-6, PMID: 24365351

[3] Li Y Teng D Shi X Qin G Qin Y Quan H . Prevalence of diabetes recorded in mainland China using 2018 diagnostic criteria from the American Diabetes Association: national cross sectional study. BMJ. (2020) 369:m997. doi: 10.1136/bmj.m997, PMID: 32345662 PMC7186854

[4] Bhandari R Sharma A Kuhad A. Novel nanotechnological approaches for targeting dorsal root ganglion (DRG) in mitigating diabetic neuropathic pain (DNP). Front Endocrinol (Lausanne). (2021) 12:790747. doi: 10.3389/fendo.2021.790747, PMID: 35211091 PMC8862660

[5] Galosi E Hu X Michael N Nyengaard JR Truini A Karlsson P. Redefining distal symmetrical polyneuropathy features in type 1 diabetes: a systematic review. Acta Diabetol. (2022) 59:1–19. doi: 10.1007/s00592-021-01767-x, PMID: 34213655 PMC8758619

[6] Weng YC Tsai SS Lyu RK Chu CC Ro LS Liao MF . Diabetic distal symmetrical polyneuropathy: correlation of clinical, laboratory, and Electrophysiologic studies in patients with type 2 diabetes mellitus. J Diabetes Res. (2020) 2020:1–11. doi: 10.1155/2020/6356459, PMID: 32695829 PMC7362296

[7] Calcutt NA. Diabetic neuropathy and neuropathic pain: a (con)fusion of pathogenic mechanisms? Pain. (2020) 161:S65–86. doi: 10.1097/j.pain.0000000000001922, PMID: 32999525 PMC7521457

[8] Fan Q Gordon SA. Recent updates in the treatment of diabetic polyneuropathy. Fac Rev. (2022) 11:30. doi: 10.12703/r/11-30, PMID: 36311537 PMC9586156

[9] Liu L Hu X Su Y Lin C Wang Y. Application and development of nanotechnology in traditional Chinese acupuncture in recent 20 years: a comprehensive review. ACS Appl Mater Interfaces. (2025) 17:22161–83. doi: 10.1021/acsami.4c22627, PMID: 40197005

[10] Schiller J Niederer D Kellner T Eckhardt I Egen C Zheng W . Effects of acupuncture and medical training therapy on depression, anxiety, and quality of life in patients with frequent tension-type headache: a randomized controlled study. Cephalalgia. (2023) 43:3331024221132800. doi: 10.1177/03331024221132800, PMID: 36622877

[11] Fan YM Li YX Zhang Y Chen D Yuan MQ Li YC . Effect of acupuncture on tic disorder: a randomized controlled clinical trial based on energy metabolomics and infrared thermography. BMC Complement Med Ther. (2024) 24:240. doi: 10.1186/s12906-024-04534-x, PMID: 38902771 PMC11191346

[12] Wen J Chen X Yang Y Liu J Li E Liu J . Acupuncture medical therapy and its underlying mechanisms: a systematic review. Am J Chin Med. (2021) 49:1–23. doi: 10.1142/S0192415X21500014 33371816

[13] Niruthisard S Ma Q Napadow V. Recent advances in acupuncture for pain relief. Pain Rep. (2024) 9:e1188. doi: 10.1097/PR9.0000000000001188, PMID: 39285954 PMC11404884

[14] Wie HS Kim SN. Therapeutic components of acupuncture stimulation based on characteristics of sensory nerve and nervous signaling pathway. J Integr Med. (2025) 23:106–12. doi: 10.1016/j.joim.2025.02.002, PMID: 40069035

[15] Lan L Wang L Sadeghirad B Tang J Liu Y Couban RJ . Acupuncture for the Management of Chronic Diabetic Peripheral Neuropathy: a systematic review and Meta-analysis of randomized controlled trials. Curr Pain Headache Rep. (2025) 29:74. doi: 10.1007/s11916-025-01386-z, PMID: 40220243

[16] Zhou L Wu T Zhong Z Yi L Li Y. Acupuncture for painful diabetic peripheral neuropathy: a systematic review and meta-analysis. Front Neurol. (2023) 14:1281485. doi: 10.3389/fneur.2023.1281485, PMID: 38046594 PMC10690617

[17] He XF Kang YR Fei XY Chen LH Li X Ma YQ . Inhibition of phosphorylated calcium/calmodulin-dependent protein kinase IIalpha relieves streptozotocin-induced diabetic neuropathic pain through regulation of P2X3 receptor in dorsal root ganglia. Purinergic Signal. (2023) 19:99–111. doi: 10.1007/s11302-021-09829-z 34973115 PMC9984656

[18] Fei X He X Tai Z Wang H Qu S Chen L . Electroacupuncture alleviates diabetic neuropathic pain in rats by suppressing P2X3 receptor expression in dorsal root ganglia. Purinergic Signal. (2020) 16:491–502. doi: 10.1007/s11302-020-09728-9, PMID: 33011961 PMC7855163

[19] Jiang Y Hu H Li X Lou J Zhang Y He X . Difference in Moxibustion-induced microcirculatory responses between the heart and lung meridians assessed by laser Doppler Flowmetry. Evid Based Complement Alternat Med. (2021) 2021:1–9. doi: 10.1155/2021/6644625 PMC8032512 33868440

[20] Tay JS Kim YJ. Efficacy of moxibustion in diabetes peripheral neuropathy. Medicine (Baltimore). (2021) 100:e28173. doi: 10.1097/MD.0000000000028173, PMID: 34889293 PMC8663870

[21] Shang J Fan W Dou Z Wu L Lu B Qian J. The efficacy and safety of warming acupuncture and moxibustion on rheumatoid arthritis: a protocol for a systematic review and meta-analysis. Medicine (Baltimore). (2020) 99:e21857. doi: 10.1097/MD.0000000000021857, PMID: 32846836 PMC7447468

[22] Chen S Liu W Liang C Liu H Wang P Fu Q. Efficacy and safety of moxibustion for knee osteoarthritis: a systematic review and meta-analysis. Complement Ther Clin Pract. (2025) 59:101979. doi: 10.1016/j.ctcp.2025.101979, PMID: 40184698

[23] Xin S Liu J Yang Z Li C. Comparative effectiveness of moxibustion and acupuncture for the management of osteoarthritis knee: a systematic review and meta-analysis. Heliyon. (2023) 9:e17805. doi: 10.1016/j.heliyon.2023.e17805, PMID: 37449100 PMC10336830

[24] Xiang W Jiang J Hu T Deng X Chen C Chen Z. The efficacy and safety of moxibustion for pressure injury: a protocol for systematic review and meta-analysis. Medicine (Baltimore). (2022) 101:e28734. doi: 10.1097/MD.0000000000028734, PMID: 35147097 PMC8830830

[25] Xu WW Teng ZQ Wan QQ Shao XM Tian HF. Herbs-partitioned Moxibustion on the navel in a rat model of primary dysmenorrhea with cold coagulation and blood stasis. J Vis Exp. (2024) 212. doi: 10.3791/66622, PMID: 39431797

[26] Yin Y Wang L Zhao L Lin L Shen X. Effect of 10.6 μm laser moxibustion on inflammation in diabetic peripheral neuropathy rats. Front Endocrinol (Lausanne). (2023) 14:1203677. doi: 10.3389/fendo.2023.1203677, PMID: 37593350 PMC10427917

[27] Dalong Z Lixin G. Guideline for the prevention and treatment of diabetes mellitus in China (2024 edition). Chin J Diabetes Mellit. (2025) 17:16–139. doi: 10.3760/cma.j.cn115791-20241203-00705

[28] Ge R Liu R He M Wu J Zhang F Huang C. The efficacy of acupuncture for diabetic peripheral neuropathy: a systematic review and meta-analysis of randomized controlled trails. Front Neurol. (2024) 15:1500709. doi: 10.3389/fneur.2024.1500709, PMID: 39758782 PMC11697586

[29] Bril V Perkins BA. Validation of the Toronto clinical scoring system for diabetic polyneuropathy. Diabetes Care. (2002) 25:2048–52. doi: 10.2337/diacare.25.11.2048, PMID: 12401755

[30] Jiang J Shen H Zhang Y Li Y Jing Y Chen X . Acupuncture treatment of diabetic peripheral neuropathy: an overview of systematic reviews based on evidence mapping. Front Neurol. (2024) 15:1420510. doi: 10.3389/fneur.2024.1420510, PMID: 39421572 PMC11483369

[31] Wang J Zhang Y Wu Q Bian Z Luo N Sun J . The efficacy and safety of electroacupuncture for diabetic peripheral neuropathy: a protocol for a systematic review and meta-analysis. PLoS One. (2024) 19:e0302228. doi: 10.1371/journal.pone.0302228, PMID: 38662762 PMC11045088

[32] Qu SY Wang HZ Hu QQ Ma YQ Kang YR Ma LQ . Electroacupuncture may alleviate diabetic neuropathic pain by inhibiting the microglia P2X4R and neuroinflammation. Purinergic Signal. (2023). doi: 10.1007/s11302-023-09972-9, PMID: 37870716 PMC12454746

[33] Zheng Y Li S Kang Y Hu Q Zheng Y Wang X . Electroacupuncture alleviates Streptozotocin-induced diabetic neuropathic pain via the TRPV1-mediated CaMKII/CREB pathway in rats. J Mol Neurosci. (2024) 74:79. doi: 10.1007/s12031-024-02256-w, PMID: 39162890

[34] Qi P Li Q Han M Cui Y Zhou X Sun Z . The analgesic mechanism of electroacupuncture at the central level for neuropathic pain: a review of studies based on animal experiments. Front Neurol. (2025) 16:1587471. doi: 10.3389/fneur.2025.1587471, PMID: 40510205 PMC12158743

[35] Ding T Sheng L Zhu H Guan H Wang Y Guo H . Efficacy and safety of external therapy of TCM for diabetic peripheral vascular disease: a protocol for systematic review and meta-analysis. Medicine (Baltimore). (2022) 101:e32362. doi: 10.1097/MD.0000000000032362, PMID: 36595808 PMC9794352

[36] Li J Hu X Liang F Liu J Zhou H Liu J . Therapeutic effects of moxibustion simultaneously targeting Nrf2 and NF-κB in diabetic peripheral neuropathy. Appl Biochem Biotechnol. (2019) 189:1167–82. doi: 10.1007/s12010-019-03052-8, PMID: 31209719 PMC6882806

[37] Singh H Chetha AS Shalikar H. Moxibustion-septic shock and necrotizing fasciitis. IDCases. (2020) 22:e00990. doi: 10.1016/j.idcr.2020.e00990, PMID: 33294368 PMC7689399

[38] Hemmi S Kurokawa K Nagai T Yokoi K Okamoto T Asano A . Relationship between the diabetic polyneuropathy index and the neurological findings of diabetic polyneuropathy. Intern Med. (2020) 59:1957–62. doi: 10.2169/internalmedicine.4499-20, PMID: 32448837 PMC7492118

[39] Zhao H Shu L Huang W Wang W Song G. Difference analysis of related factors in macrovascular and microvascular complications in Chinese patients with type 2 diabetes mellitus: a case-control study protocol. Diabetes Metab Syndr Obes. (2019) 12:2193–200. doi: 10.2147/DMSO.S213848, PMID: 31695462 PMC6814870

[40] Zheng H-D Wang Z-Q Li S-S Lu Y Huang Y Zhou C-L . Effect of acupoints on acupuncture-moxibustion and its therapeutic mechanism. World J Tradit Chin Med. (2020) 6:239–48. doi: 10.4103/wjtcm.wjtcm_18_20

[41] Ma S Huang H Xue F Wang Q Yu S Hou Q . Acupoint prescriptions, treatment protocol and outcome evidence for acupuncture in diabetic peripheral neuropathy: a scoping review of clinical studies. Eur J Integr Med. (2024) 70:102376. doi: 10.1016/j.eujim.2024.102376

[42] Tan Y Hu J Pang B Du L Yang Y Pang Q . Moxibustion for the treatment of diabetic peripheral neuropathy: a systematic review and meta-analysis following PRISMA guidelines. Medicine (Baltimore). (2020) 99:e22286. doi: 10.1097/MD.0000000000022286, PMID: 32991431 PMC7523832

[43] Galiero R Caturano A Vetrano E Beccia D Brin C Alfano M . Peripheral neuropathy in diabetes mellitus: Pathogenetic mechanisms and diagnostic options. Int J Mol Sci. (2023) 24:3554. doi: 10.3390/ijms24043554, PMID: 36834971 PMC9967934

[44] Dietzel J Horder S Habermann IV Meyer-Hamme G Hahn K Ortiz M . Acupuncture in diabetic peripheral neuropathy-protocol for the randomized, multicenter ACUDPN trial. Trials. (2021) 22:164. doi: 10.1186/s13063-021-05110-1, PMID: 33637134 PMC7907791

